# Therapeutic Modulation of the Complement System in Kidney Transplantation: Clinical Indications and Emerging Drug Leads

**DOI:** 10.3389/fimmu.2019.02306

**Published:** 2019-10-01

**Authors:** Vasishta S. Tatapudi, Robert A. Montgomery

**Affiliations:** NYU Langone Transplant Institute, New York, NY, United States

**Keywords:** kidney transplant, complement–immunological term, HLA, monoclonal Ab, antibody mediated allograft rejection

## Abstract

The complement system is integral to innate immunity, and it is an essential deterrent against infections. The complement apparatus comprises of >30 fluid-phase and surface-bound elements that also engage with the adaptive immune system, clear harmful immune complexes, and orchestrates several salutary physiological processes. An imbalance in the complement system's tightly regulated machinery and the consequent unrestrained complement activation underpins the pathogenesis of a wide array of inflammatory, autoimmune, neoplastic and degenerative disorders. Antibody-mediated rejection is a leading cause of graft failure in kidney transplantation. Complement-induced inflammation and endothelial injury have emerged as the primary mechanisms in the pathogenesis of this form of rejection. Researchers in the field of transplantation are now trying to define the role and efficacy of complement targeting agents in the prevention and treatment of rejection and other complement related conditions that lead to graft injury. Here, we detail the current clinical indications for complement therapeutics and the scope of existing and emerging therapies that target the complement system, focusing on kidney transplantation.

## Introduction

**Figure 1:**
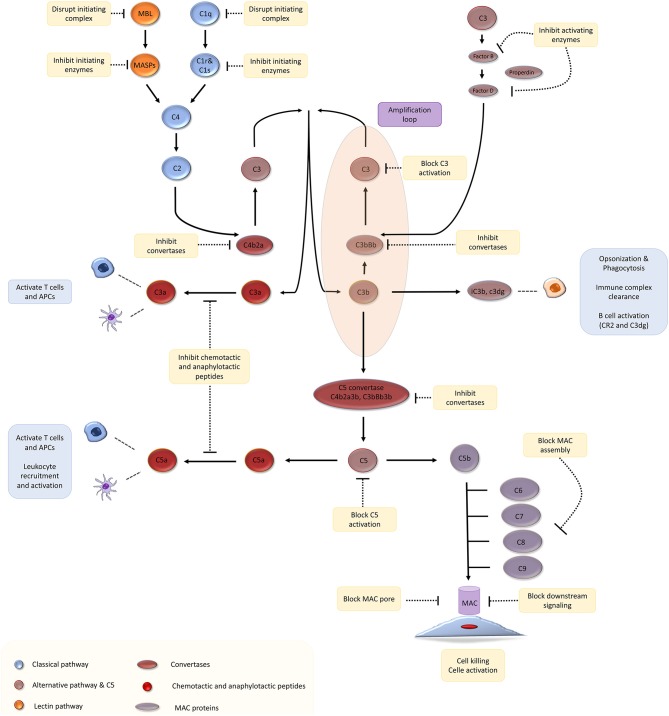
The complement cascade and potential therapeutic targets. Adapted with permission from Morgan and Harris (4).

Complement proteins account for 3 g/l of plasma and make up ~15% of the globulin fraction (1). The complement system is activated via three canonical pathways: (Figure 1) (a) the classical pathway, triggered by recognition of subclasses of surface-bound IgG and IgM antibodies by complement component C1q; (b) the lectin pathway, triggered by recognition of bacterial surface sugars by mannose-binding lectin (MBL); and (c) the alternative pathway that is constitutively active due to spontaneous hydrolysis of C3, a phenomenon that has been christened C3 “tickover” (2). These three pathways lead to the formation of critical enzymes complexes called C3 convertases, which trigger events that culminate in the generation of the cell-killing membrane attack complex (MAC) (1, 3). We direct you to recent reviews on complement biology for a detailed description of the pathways and mechanism of complement activation and regulation (3–6).

The approval of the complement-targeting anti-C5 antibody, eculizumab, by the United States Food and Drug Administration (FDA) for the rare disease paroxysmal nocturnal hemoglobinuria (PNH) in 2007 has led to a renaissance in complement therapeutics (7). PNH is a hematological disease characterized by transfusion-dependent hemolytic anemia and life-threatening thrombosis. PNH results from the clonal expansion of hematopoietic stem cells that have a somatic mutation in a gene that is critical to the synthesis of glycosylphosphatidylinositol (GPI) (8). GPI serves as an anchor for many proteins to the cell surface, including CD59, a MAC regulatory protein (8, 9). Therefore, red cells deficient in GPI are susceptible to complement-mediated intravascular hemolysis. PNH is an uncommon disease that affects 10,000 people in North America and Western Europe (8). Untreated, it is associated with a dismal median survival of 10 years after diagnosis (8). Eculizumab (Soliris, Alexion) is a recombinant humanized anti-C5 monoclonal antibody that prevents the cleavage of C5 by C5 convertase. In a phase 3 clinical trial published in 2006, eculizumab stabilized hemoglobin levels in transfusion-dependent patients with PNH (9). Following this trial, the FDA approved the use of eculizumab for PNH in March 2007.

In atypical hemolytic uremic syndrome (aHUS), complement overactivity leads to systemic thrombotic microangiopathy involving the kidney, central nervous system, heart, and gastrointestinal tract. In 2011, the FDA approved eculizumab for use in aHUS following a clinical trial demonstrating significant improvement in renal function with its use (10). Hereditary angioedema (HAE) is an inherited disorder in which a deficiency of C1-inhibitor (C1-INH) leads to dysregulation of the complement cascade and the kallikrein pathway leading to recurrent episodes of life-threatening angioedema. C1-INH is a serine protease inhibitor (SPI) that removes activated C1r and C1r from C1q (4). Two nano-filtered C1-INH products derived from human serum, cinryze (Takeda/Shire Pharmaceuticals) and berinert (CSL Behring), were approved for the treatment and prophylaxis of attacks of HAE after randomized trials demonstrated their efficacy (4, 11). The clinical success of these two drugs has fueled interest in complement and sparked the development of complement targeting therapeutics for numerous other conditions like age-related macular degeneration (AMD), C3 glomerulopathy, and an array of conditions related to kidney transplantation.

Kidney transplantation is the therapy of choice for eligible patients with end-stage renal (kidney) failure. However, mismatches in human leukocyte antigens (HLA) between the donor and the recipient can cause the recipient's immune system to reject the transplanted allograft. The mechanism of immune injury in rejection has been the subject of intense research over the last few decades. This has led to a detailed understanding of the molecular processes involved in rejection, and it is now clear that the complement system plays a central role, especially in the pathogenesis of antibody mediated rejection (AMR). Several groups have recently published results of clinical trials exploring the role of complement blockade in AMR of transplant kidneys.

## Role of Complement in Kidney Transplantation

Kidney transplantation is the treatment of choice for end-stage renal disease (ESRD) due to the superior long-term survival, cost savings to the health care system, and the greater quality of life it offers relative to dialysis (12, 13). However, the prevention and treatment of rejection is still a major impediment to successful transplantation (14). The transplantation of tissues from a donor who is genetically disparate from the recipient elicits an immune response in the recipient against alloantigens that, if uncontrolled, can lead to allograft destruction (13).

**Figure 2:**
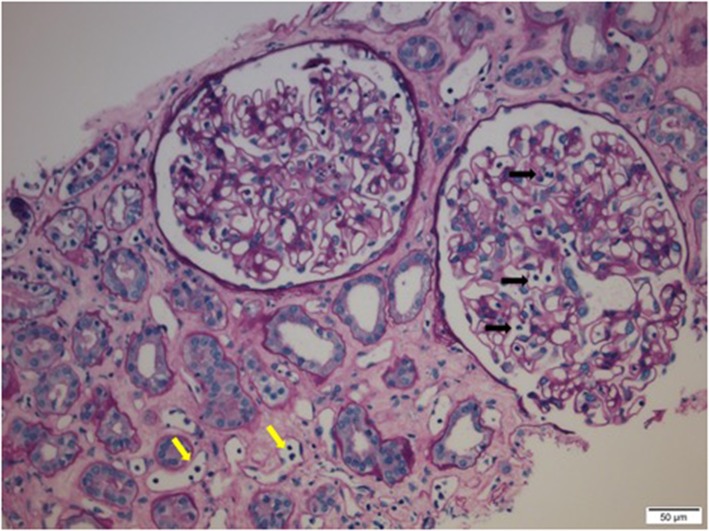
Infiltrating polymorphonuclear leukocytes (PMNs) in renal glomerular capillary loops (black arrows) and peritubular capillaries (yellow arrows) in a renal allograft undergoing acute antibody mediated rejection (AMR).

**Figure 3:**
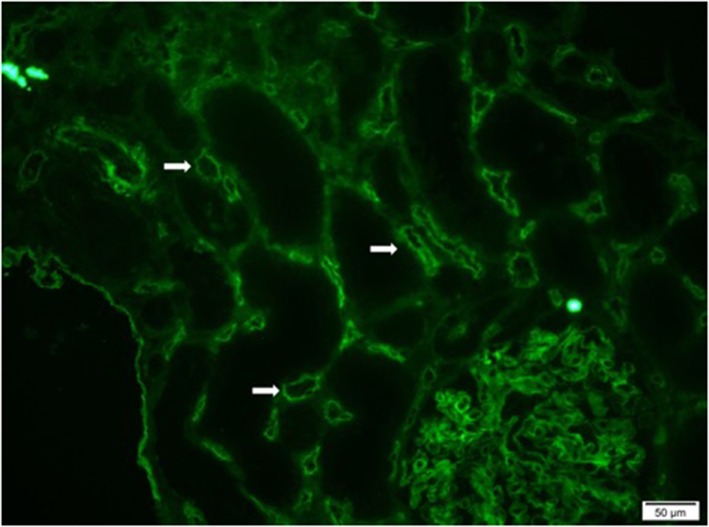
Immunofluorescence microscopy demonstrating diffuse and prominent deposition of C4d on the endothelium of peritubular capillaries (white arrows) in a renal allograft undergoing acute antibody mediated rejection (AMR).

Preformed circulating anti-HLA antibodies are present in up to 30% of patients awaiting kidney transplantation as a result of previous exposure to allo-HLA antigens from blood transfusions, pregnancies, and previous transplants and may cause immediate graft failure due to hyperacute rejection (15). The incidence of acute allograft rejection has declined significantly since the emergence of calcineurin inhibitors (CNIs) in the 1980s (16). However, this has not resulted in a concurrent improvement in long-term allograft survival (17). Late-onset AMR has emerged as a leading cause of allograft loss and is increasingly recognized as the reason for the poor long-term graft survival (18). In AMR, donor-specific antibodies (DSAs) bind to mismatched HLA molecules and can trigger classical complement pathways leading to allograft vascular injury (19). This injury is mediated by the MAC as well as C3a and C5a driven inflammation (19). Histologically, this is characterized by the demonstration of infiltrating polymorphonuclear leukocytes (PMNs) and macrophages in renal glomerular and peri-tubular capillaries (Figure 2). Complement split product, C4d, is prominently deposited in the microvasculature of renal allografts undergoing AMR (Figure 3) (20). C4d remains covalently attached to the endothelium and is detected in peritubular capillaries in renal allograft biopsies by immunohistochemistry. It acts as a footprint of HLA-binding by DSAs and resultant complement activation (21).

The advent of sensitive solid-phase Luminex platform bead-based assays for the detection of HLA antibodies has enhanced our ability to detect DSAs and diagnose AMR (22, 23). In this flow-cytometry based assay, fluorochrome impregnated micro-beads coated with HLA proteins are incubated with patient serum. Anti-HLA antibodies bind the cognate HLA antigens on the beads and are detected by a dual- laser instrument (24). In 2003, standardized diagnostic criteria for AMR that featured C4d microvascular deposition and the presence of circulating DSAs were incorporated into the classification schema for kidney transplant pathology set forth by the Banff group (25). Spurred by the success of complement therapeutics in diseases mediated by complement dysregulation like PNH and aHUS, the role of pharmacologic complement blockade is being explored through clinical trials in a variety of kidney transplant-related settings (26).

The presence of preexisting DSA increases the risk of AMR and may adversely impact the graft survival even in recipients of negative lymphocytotoxic cross-match kidney transplants (23). C1q-binding DSA appear to be associated with significantly lower long-term graft survival than non-complement fixing DSA, indicating that the complement activation by DSA is critical to their pathogenic potential (25). Characterizing the pathogenic potential of DSA detected by modern sensitive assays is valuable in the risk stratification of patients with respect to the perils of AMR. This will allow transplant physicians to adopt a more nuanced approach to the utilization of potent and frequently expensive immunosuppressive agents, and thereby balance the risk of rejection with that of debilitating infections and malignancies.

## Complement Therapeutics in Kidney Transplantation

### Complement Modulation in Treatment of AMR

Enhanced understanding of the etiology of AMR and the prominent role played by the complement system implies a possible role for pharmacologic complement inhibition in clinical transplantation. The terminal complement inhibitor, Eculizumab, and C1 esterase inhibitor (C1-INH) have been the subjects of recent clinical trials exploring the potential of targeting the complement cascade in the prevention and treatment of AMR (19, 27, 28).

DSA depletion by plasmapheresis combined with intravenous immunoglobulin (IVIg) is currently the mainstay of treatment for AMR (29). However, regimens reported in literature differ broadly with respect to the duration of therapy, dosing of IVIg, and the role of B-lymphocyte and plasma-cell targeting agents like rituximab and bortezomib (26, 30). Based on promising single center experience treating acute AMR, a multi-center trial that began enrollment in 2013 evaluated the role of eculizumab in the treatment of biopsy-proven AMR and acute graft dysfunction (31–33). Patients in the standard of care (SOC) arm received three sessions of plasmapheresis with IVIg administered after each session. Those in the treatment arm received a first dose of 1,200 mg of eculizumab followed by 4 weekly doses of 900 mg and a dose of 1,200 mg at week 5 (31). Patients received additional doses of 1,200 mg at weeks 7 and 9 if DSA levels at week 6 remained >50% of baseline (31). However, recruitment for this trial was terminated in 2017 due to the study drug's failure to improve allograft function assessed by estimated glomerular filtration rate (eGFR) 3 months after transplantation (31).

In AMR, activation of the classical pathway is triggered by ligation of the C1 complex to HLA-antigens that are bound by DSA. Therefore, complement inhibitors targeting C1 can be leveraged to treat AMR while sparing the alternative pathway, thereby potentially averting infectious complications (34). Additionally, complement inhibition upstream of C5 with C1 inhibition would be predicted to block the generation of complement activation products of C3 that contribute to neutrophil and monocyte recruitment and tissue injury in AMR. Two nano-filtered C1-INH products derived from human serum, cinryze (Takeda/Shire Pharmaceuticals) and berinert (CSL Behring), are approved for the treatment and prophylaxis of attacks of HAE. The role of C1-INH in the treatment of AMR was explored by two recent studies (27, 28). Viglietti et al. treated six patients who had AMR that was not responsive to SOC therapy with C1-INH (berinert, CSL Behring) and high-dose IVIg for a duration of 6 months (27). At the end of the 6-month follow-up period, the authors reported significant improvement in mean eGFR. Fewer patients demonstrated C4d deposition on allograft biopsies and circulating C1q fixing DSAs. However, other histologic features, like chronic glomerulopathy, were unchanged (27). Montgomery et al. conducted a randomized phase 2 trial to evaluate the role of a 2-week course of C1-INH (Cinryze, Shire Pharmaceuticals) as an add-on therapy to SOC plasmapheresis and low-dose IVIg, compared to SOC alone. Nine patients were randomized to each arm. While there was no significant difference in graft survival and histologic findings of AMR after 20 days, 6-month allograft biopsies showed an absence of transplant glomerulopathy in the C1-INH treated patients, whereas three out of seven patients in the placebo arm were found to have this histologic feature that is associated with chronic antibody mediated injury and premature allograft loss (28). It is to be noted that the above two studies differed in study design and the C1-INH product used (27, 28). Both of these C-INH products are also the subject of recent phase 3 trials. A double-blind, randomized, placebo-controlled study (NCT03221842) of human plasma-derived C1-INH, berinert (CSL Behring), as an add-on to SOC for the treatment of refractory AMR in adult kidney transplant recipients, is currently underway. The investigators aim to enroll 90 study subjects and measure the primary outcome of time to loss of treatment response (35). However, another multicenter, randomized, placebo-controlled study (NCT02547220) to evaluate the efficacy and safety of the other C1-INH product, cinryze (Takeda/Shire Pharmaceuticals), for the treatment of acute AMR in kidney transplant recipients, was terminated early because interim analysis showed that the study met the pre-specified criteria for futility (36). Therefore, the jury is still out on the utility of C1-INH in the treatment of AMR.

C1 complex is a multi-molecular cluster constituted by the PRP, C1q, and two serine proteases, C1r, and C1s (4). Upon activation, C1s proceeds to cleave C4 and C2 and results in the formation of C4b and C2a, respectively (4). These activation fragments associate to form the C4b2a complex, which is the classical pathway's C3 convertase (4, 26). An anti-C1s murine monoclonal antibody TNT003 and its humanized variant BIVV009 have been shown to block chemotaxis of macrophages in experimental models of HLA antibody-triggered classical pathway activation (37, 38). Eskandary et al. published the findings of a phase 1 cohort study of 10 kidney transplant recipients with acute or chronic active AMR (median of 4.3 years post-transplant) who received BIVV009 (34). Patients enrolled had features of classical pathway activation like complement-fixing DSA or C4d staining in peritubular capillaries in allograft biopsies. No severe adverse events were reported in 7 weeks of follow up. C4d staining turned from positive to negative in five patients and a significant improvement in C4d deposition in biopsies of two other patients. However, microvascular inflammation, gene expression profiles, DSA strength, and renal allograft function were unchanged (34). While BIVV009 appeared to be well-tolerated, it needs to be studied in future trials to determine its role in AMR therapy.

### Complement Modulation in Highly Sensitized Kidney Transplant Candidates

Sensitization to HLA is often a significant challenge to kidney transplantation (39). The calculated PRA (CPRA) score estimates the population prevalence of potential donors with HLA antigens against whom a transplant candidate is sensitized (24, 40). Approximately one-third of kidney transplant candidates awaiting a deceased donor transplant in the US have a CPRA >30%, and this decreases the likelihood that they will receive an organ offer (41). These patients have a prolonged wait time for transplantation and a reduced transplant rate (42). While policies that preferentially assign allocation points to patients who are highly sensitized, and innovative strategies like kidney paired donation may facilitate transplantation in this patient population, many–especially those with a CPRA >99%–are unlikely to receive a transplant. In such patients, transplantation across HLA barriers by “desensitization” may be their only chance at emancipation from dialysis (39). Living donor kidney transplantation after desensitization by the depletion of DSA is associated with a greater survival rate than waiting for an HLA compatible kidney transplant or staying on dialysis (42, 43). However, there is a high incidence of AMR in recipients of HLA incompatible kidney transplants following desensitization by the current approaches that employ plasmapheresis and IVIg, with some centers reporting an AMR rate as high as 40%. AMR, in this setting, can be difficult to treat and result in graft loss (44).

Eculizumab's role in the prevention of AMR in HLA-incompatible kidney transplants was tested in an observational study of 26 highly sensitized patients by Stegall et al. (45). The rate of AMR in the study group was compared to 51 historical controls. Study participants received plasmapheresis before transplant if the strength of the B-cell flow-cytometry crossmatch with their respective living donors was >300 mean channel shifts (MCS) in order to lower their DSA to acceptable levels for transplantation. The eculizumab regimen consisted of two doses given peri-operatively, followed by weekly doses for 4 weeks. Further dosing depended on assessment of DSA levels thereafter. In contrast, those in the historic control group had received pre-transplant plasmapheresis and an additional 4–14 post-transplant plasmapheresis treatments. In this study, recipients who received preemptive treatment with eculizumab, had a 7.7% incidence of AMR by 3 months post-transplant, whereas the historic control group had a significantly higher incidence of AMR (41.2%) (45). However, long-term follow-up demonstrated that histologic findings such as microvascular inflammation, transplant glomerulopathy, and C4d deposition were no different between the two groups. Among subjects who had sustained levels of DSA, eculizumab treatment did not prevent transplant glomerulopathy. This may be representative of events upstream of the activation of the terminal complement pathway, like anaphylatoxin C3a and iC3b, C3dg opsonins which drive chronic injury and inflammation. Additionally, injury can occur through complement-independent mechanisms such as Fc-mediated antibody dependent cellular cytotoxicity by NK cells, or the direct activation of endothelial cell signaling pathways by DSA AMR (46).

Contrary to the encouraging findings from these non-randomized studies reported by Stegall et al., a randomized trial of eculizumab in the prevention of AMR in HLA-sensitized recipients of living-donor kidney transplants initially failed to show any benefits (47). In this multicenter study, 102 HLA-incompatible candidates were desensitized prior to transplant with SOC therapy (plasmapheresis and intravenous immunoglobulin). At the time of transplant they were randomized to either receive post-transplant eculizumab or additional plasmapheresis. There was no difference in the primary composite outcome which included histologically-proven AMR, graft loss, patient death, or loss to follow-up 9 weeks after transplant (5/51 vs. 7/51, *P* = 0.76) (47).

*Post-hoc* analysis demonstrated that the failure to show benefits in the treatment arm was likely related to trial design, inasmuch as there was discordance between the central and local pathology assignment of the diagnosis of AMR. When the central pathologists re-analyzed the biopsies with the clinical data that had been available to the local pathologists, the discordance went away, and the difference between the treatment and control groups became significant. Likewise, if all categories of AMR severity were assigned the diagnosis of AMR, the results were also significant. Most clinicians believe that the diagnosis of AMR is binary, and the grading system used in the study was not clinically relevant, again reinforcing a positive result to the study (48). Another *post-hoc* analysis, showed the peril of changing the DSA strength inclusion criteria mid-study which was done to boost enrollment, allowing patients with relatively low immunologic risk for AMR (including patients with C1q negative DSA) to enter the trial. This had the effect of lowering rates of AMR in both study arms below expected based on prior reports in HLA incompatible transplant recipients (49).

C1-INH is a promising therapeutic target in transplantation. Its role in the prevention of AMR in HLA-incompatible transplantation was evaluated in a phase 1/2, placebo-controlled study by Vo et al. (50). The study randomized 20 patients who underwent kidney transplantation following desensitization with a regimen consisting of IVIg, rituximab, and plasmapheresis into two arms. The C1-INH group received one dose intraoperatively, followed by a twice-weekly regimen for a total of seven doses (50). Delayed graft function (DGF) occurred in only one patient treated with C1-INH and in four patients in the placebo group, suggesting that C1-INH may offer protection against ischemia-reperfusion injury. AMR occurred in none of the C1-INH treated patients, and in one of the placebo-treated patients during the study period. There appeared to be a lower incidence of C1q-binding DSA in the C1-INH group. No serious treatment-related adverse effects were reported in either group (50). The question of whether complement inhibition has a role in the prevention of acute or chronic AMR in highly sensitized patients undergoing HLA incompatible transplantation remains an open question which can only be answered by well-designed large randomized trials. Complement inhibition upstream of C5 with C1-INH is indeed an attractive, logically sound target, since it prevents the formation of anaphylatoxins and opsonins (4).

### Complement Modulation to Prevent Recurrence of Disorders of Complement Dysregulation Post-transplant

Alternative complement pathway dysregulation underlies the etiopathogenesis of two ultra-rare kidney diseases, aHUS, and C3 glomerulopathies, both of which can lead to ESRD (51). Genetic abnormalities implicated in the causation of these conditions include mutations in complement factor H, complement factor I, membrane cofactor protein (MCP), complement factor B, and C3. In 2011, the FDA approved eculizumab for aHUS therapy following a clinical trial that demonstrated that it significantly improved kidney function (10). While there is no approved therapy for the treatment of C3 glomerulopathy, in a retrospective study conducted by Avasare et al., a combination of anti-proliferative agent mycophenolate mofetil (MMF) and corticosteroids induced remission in 67% of patients (52). At this time, the global nephrology professional society guidelines state that there is not enough evidence to support eculizumab's role as primary therapy for rapidly progressive C3 glomerulopathy (53). An unfortunate aspect of the natural history of both of these diseases is that they recur even after successful kidney transplantation (54). Apart from its well-established role in the treatment of aHUS in native kidneys, terminal complement blockade with eculizumab is increasingly recognized as an effective therapy for prevention and treatment of aHUS after renal transplantation (51, 55). In the case of C3 glomerulopathy, however, the role of eculizumab has not been clearly established. Evidence from small case series does not support the use of eculizumab in this condition, either in native or transplanted kidneys, except perhaps in cases where soluble or serum MAC (sMAC) is elevated (56). Small phase one clinical trials have been initiated to test the safety of C3 inhibitors in the treatment and prevention of C3 glomerulopathy (57).

## Complement in Xenotransplantation

Outcomes in transplantation are hindered by drug-related adverse effects, chronic allograft rejection, and a scarcity of organs (58). Currently, more than 110,000 individuals are awaiting a transplant in the US; only a third of these receive a transplant, and many die waiting for an organ (59). Xenotransplantation entails transplantation of organs across species, and when combined with induction of tolerance, it could be the answer to the above issues (60, 61).

Transplantation of organs derived from pigs is an option that has attracted the greatest interest in the field (60). However, pigs express an epitope called α-1,3 Gal (62). This is a terminal carbohydrate modification of many glycoproteins and glycolipids that is present in most species and is made by an enzyme called α-1,3-Gal transferase (58). Old world monkeys and humans lack this epitope due to a mutation in the 1,3-Gal transferase gene. Since many species like bacteria and other microbes do have the α-1,3-Gal epitope, humans are exposed to it, and we have naturally occurring antibodies circulating that recognize α-Gal (62). When organs are transplanted from pigs to primates, these antibodies bind the donor vascular endothelium to activate complement and may cause hyper-acute rejection (HAR) or delayed xenograft rejection (DXR) (62). In the early 2000s, the development of technology to knock out the gene for α-1,3-Gal transferase accelerated the tempo of research in the field of xenotransplantation (63).

With advances in CRISPR (clustered regularly interspaced short palindromic repeat)/CRISPR-associated 9 (Cas9) technology, various groups have created genetically altered pigs to facilitate xenotransplantation (58). In addition to knocking out pig glycan genes, human complement regulatory proteins have been expressed from transgenes in some genetically engineered pigs, and is seen as an important strategy to prevent xenograft injury. In 1998, White et al. reported a median graft survival of 40 days in hearts of transgenic pigs expressing human decay accelerating factor (hDAF/CD55) that were transplanted into cynomolgus monkeys or baboons. These primates received potent immunosuppression with cyclosporine, cyclophosphamide, and steroids. This group expressed hope at the time that the use of organs from transgenic pigs may help to solve the problem of donor shortage in clinical transplantation (64). Pigs expressing CD46, CD55, and CD59 on the surface of vascular endothelial cells have been used in preclinical primate studies and appear to extend the xenograft survival (65, 66).

More recently, progress has been made by combining multiple glycan knockouts with human complement and coagulation regulatory gene knock ins (67). In 2016, Mohiuddin et al. published findings describing prolonged graft survival following pig to primate heart transplantation using immunosuppression and genetic engineering (68). Their study involved α-1,3-Gal transferase knockout pigs expressing human complement regulatory protein CD46 and human thrombomodulin. Pig hearts were heterotopically transplanted in the abdomen of baboons. Immunosuppression included induction with anti-thymocyte globulin and anti-CD20 antibody, maintenance with mycophenolate mofetil, and high dose anti-CD40 antibody. The authors reported a median graft survival of 298 days and a longest graft survival of 945 days in five consecutive recipients using this regimen. However, lowering the dose of anti-CD40 antibody at day 100 or after 1 year led to a rebound of anti-pig antibodies and graft loss (68). More recently life-supporting function of baboon orthotopic heart xenografts have been shown to consistently exceed 195 days, and this has been seen as a major breakthrough (69).

While currently available, transgenic knockout pigs may provide a solution to the problem of potent and immediate humoral responses due to pre-formed anti-Gal antibodies and allow transplantation. However, agents utilized in most of the successful preclinical primate studies do not have a track record of safety in humans. Therefore, to realize the goal of clinical xenotransplantation with sustained graft function in humans, tolerance induction is seen as a key element.

## Challenges and Future Directions

Severe infections of *Neisseria meningitidis* and other encapsulated bacteria like *Streptococcus pneumoniae* have been well-documented in people with inherited deficiencies in complement components (7). The nature of the deficiency determines the specific infection to which affected individuals are susceptible. For example, abnormalities of the terminal complement pathway predispose individuals to *N. meningitidis* infections, whereas C3 deficiency leads to a broad array of infections (7). Given its crucial role in immunity, prolonged pharmacologic complement blockade will likely involve an incremental increase in infectious risk above what we have become accustomed to with traditional immunosuppression. Another important consideration is the remarkably high cost of complement-targeting therapeutics that are currently approved. A market analysis of therapeutic agents available for the treatment of PNH conducted in 2007 estimated the average annual cost per patient receiving eculizumab, a drug with orphan disease designation, is $389,000 (8). This puts pressure on health care systems and makes the drug inaccessible in many regions in the world (7).

Several complement inhibitors are currently under development for a variety of disorders involving complement dysregulation. Compstatin is a peptide agent that prevents cleavage of C3 by C3 convertase (4). The FDA granted orphan drug status to Compstatin analog, AMY-101 (Amyndas Pharmaceuticals SA) for use in PNH and C3 glomerulopathy^1^^,^^2^. In the future, the availability of targeted therapeutic agents that interfere with the complement cascade at various levels will provide valuable opportunities to mitigate allograft injury, not just due to AMR, but also from ischemia-reperfusion injury and recurrent complement mediated glomerulopathies. Experience in kidney transplantation with these agents can also be potentially translated into successful therapies for heart, lung, and liver transplant recipients.

## Author Contributions

All authors listed have made a substantial, direct and intellectual contribution to the work, and approved it for publication.

### Conflict of Interest

RM received research grants from Alexion (manufacturer of Soliris–Eculizumab) and Shire ViroPharma; served as a paid consultant for Alexion, Shire ViroPharma and CSL, Behring; and received travel honoraria from Alexion and Shire VioPharma during the conduct of this study. He served on advisory boards for Genentech Scientific/ROCHE, True North/iPerian, Novartis, and Hansa Medical; received consulting fees from OrbidMed, GuidePoint Global, Sucampo, Astellas, and Shire; and received research grants from Immune Tolerance Network, ViroPharma, Hansa and Alexion. The remaining author declares that the research was conducted in the absence of any commercial or financial relationships that could be construed as a potential conflict of interest.

## Footnotes

^1^https://www.fiercepharma.com/pharma/amyndas-lead-candidate-amy-101-receives-orphan-drug-status-from-fda-and-ema-for-treatment-c3

^2^https://www.fiercebiotech.com/biotech/fda-grants-orphan-drug-designation-for-pnh-to-amyndas-pharmaceuticals-novel-complement
